# Microsurgical Reconstruction with Free Tissue Transfer in Skin Cancer Patients: A Systematic Review

**DOI:** 10.3390/cancers17142371

**Published:** 2025-07-17

**Authors:** Tito Brambullo, Stefano L’Erario, Francesco Marena, Roberta Carpenito, Alfio Luca Costa, Vincenzo Vindigni, Franco Bassetto

**Affiliations:** Plastic Surgery Unit, Department of Neurosciences, University of Padua, 35122 Padua, Italy; stefano.lerario@aopd.veneto.it (S.L.); francesco.marena@aopd.veneto.it (F.M.); roberta.carpenito@aopd.veneto.it (R.C.); alfiolucacosta.alc@gmail.com (A.L.C.); vincenzo.vindigni@unipd.it (V.V.); franco.bassetto@unipd.it (F.B.)

**Keywords:** basal cell carcinoma, free flaps, free tissue transfer, melanoma, microsurgery, skin cancer, squamous cell carcinoma, surgery

## Abstract

Background/Objectives: The gold standard of treatment for both melanoma and non-melanoma skin cancers is wide surgical resection to obtain oncological radicality, which occasionally results in functional or aesthetic impairment, potentially affecting quality of life. Despite the increased complexity of the technique, extended duration of hospitalization, and prolonged surgical operative times, microsurgery can facilitate the reconstruction of locally invasive skin cancers following ablative surgery and may yield superior functional and aesthetic outcomes. Consequently, microsurgical reconstruction is more likely to be necessary if a large skin tumor requires excision. However, the impact of this extensive and complex procedure on patients with skin cancer has not yet been fully elucidated. The objective of this research was to critically analyze the utilization of free flap reconstruction subsequent to skin cancer therapy. Through a comprehensive examination of published data, this study aimed to assess the potential benefits and drawbacks associated with this reconstructive approach. Methods: A systematic review of studies that were published from January 2004 to May 2024 was conducted using the MEDLINE online database search. To present an evidence summary and provide a systematic approach and quality assessment, the GRADE^®^ rating was applied to the results. Results: This review summarizes the oncological and clinical data, including previous interventions, adjuvant and neoadjuvant therapies, nodal status, distant metastasis, and follow-up time. Surgical outcome parameters such as healing time, flap survival, revision rate success, and minor and major complications were documented. Along with the findings, a quality assessment of the studies was also provided. Conclusions: This systematic review underscores the extensive use and efficacy of microsurgery for reconstruction after skin cancer excision; however, the literature remains limited by inconsistent reporting of oncological outcomes and the lack of a standardized approach to evaluate the impact of free flap reconstruction on both immediate and long-term cancer-specific results.

## Simple Summary

Surgery is still a fundamental step in the treatment of melanoma and non-melanoma skin tumors; however, it must involve radical removal of the tumor. In the context of locally advanced skin cancer, this approach may require autologous tissue transfer using a microsurgical technique to repair the defect. This study aimed to investigate the oncological impact of reconstructive microsurgery in patients with skin cancer by examining literature data through a systematic review. The analysis revealed that microsurgical reconstruction is widely used after skin cancer resection, with consistent results in terms of effectiveness and a low rate of complications; however, a broad gap of data still persists regarding the survival rates and the relationship between tumor burden and reconstructive choice.

## 1. Introduction

Melanoma skin cancers (MSCs) and non-melanoma skin cancers (NMSCs) are the most common types of cancer [1], significantly impacting both quality and duration of life. It is estimated that one in five Americans will develop one or more skin cancers during their lifetime [2]. NMSCs include a variety of skin cancers, the most prevalent being basocellular carcinoma (BCC) and squamocellular carcinoma (SCC) [3], with an annual incidence of about 6.3 million new cases, which, although underestimated [4], accounts for about 1/3 of all cancers diagnosed worldwide every year [5]. Both BCCs’ and SCCs’ epidemiological trends show an increase in incidence over the last few decades, with BCC incidence increasing by 145% and SCC incidence increasing by 263% between 1976–1984 and 2000–2010 [6]. Melanoma skin cancer represents 1.8% of all new cancer diagnoses, with a yearly incidence of 25 new cases per 100,000 population in Europe, 30 cases per 100,000 population in the USA, and 60 cases per 100,000 population in Australia and New Zealand [7]. Like NMSCs, epidemiological trends also show an increase of 2 to 4-fold in incidence of invasive MSCs over the last few decades; however, from the mid-1990s and early 2000s, the incidence of thin melanoma (with Breslow thickness below 1 mm) has been decreasing in young adults, as a result of efficient prevention programmes [7]. The gold standard of treatment for both NMSCs and MSCs, when operable, is wide surgical resection allowing negative histopathological margins and oncological radicality. It has been shown that complete removal rates for low-risk BCCs with a surgical excision of 4 mm clinical margins reach up to 95%, whereas high-risk BCCs require wider surgical margins or surgical techniques that allow intraoperative margin assessment, like Mohs [8]. For cSCCs, the NCCN guidelines also recommend for high-risk tumors (located in high-risk zones or with other high-risk histologic features) margins of at least 4 mm, 6 mm, and 9 mm for lesions with a diameter < 1 cm, between 1 to 1.9 cm, and >2 cm, respectively [9]. In any case, reconstruction is not recommended until negative margins are confirmed, not to alter the architectural structure of the tissue. In regards of MSCs, AAD and NCCN recommend wide excision with negative margins of 0.5 to 1 cm for in situ melanomas (Level II/III, Grade A), 1 cm margins for tumors with a Breslow thickness < 1 mm (Level I/II, Grade A), 1 to 2 cm margins for tumors 1 to 2 mm thick (Level I, Grade A), and 2 cm margins for melanomas thicker than 2 mm (Level I, Grade A) [10].

Sentinel lymph node biopsy (SLNB) is recommended for Breslow thickness > 0.8 mm, and should be considered in pT1a patients with high-risk factors (young age, high mitotic rate, lymphovascular invasion, positive margins on biopsy) [10] that increase the risk of a positive sentinel node above 5% [11,12]. Surgery may have a role in different steps of skin cancer treatment, from diagnostic incisional biopsies and wide curative excisions to palliative debulking of advanced-stage cancers. It is even associated with systemic chemotherapeutic or immunotherapeutic treatment, both in a neoadjuvant or adjuvant setting. Wide resections to obtain oncological radicality, however, may lead to wide defects or functional or aesthetic impairment, affecting quality of life. Therefore, reconstruction must aim, once histopathological examination confirms radicality, to restore function and guarantee adequate soft tissue coverage and pleasant appearance. According to the concept of “reconstructive toolbox” [13], there are several surgical techniques that could be chosen when planning the reconstructive time in order to obtain the best possible outcome for each patient. Split-thickness skin grafts, local or locoregional flaps, and acellular dermal matrices are some of the surgical techniques often employed to reconstruct small to medium-sized defects in different anatomic regions. When adequate coverage or optimal functional and aesthetic results are not achievable through the aforementioned techniques, however, microsurgical reconstruction should be considered [14]. In spite of the superior complexity of the technique, longer time of stay and surgical operative times, microsurgery can allow the reconstruction of locally invasive skin cancers after demolitive surgery and may determine better functional and aesthetic outcomes. Another benefit of microsurgical tissue transfer techniques is that they enable oncologic surgeons to attain free margins, even in regions that are challenging to reconstruct, such as the head, neck, or extremities. In these areas, the limited availability of neighboring soft tissues often impedes repair of the defect, resulting in less-than-optimal excision [15]. Consequently, if a large tumor or infiltrative component is detected at the first observation, microsurgical reconstruction is more likely to be required. However, the impact of these long and complex procedures on skin cancer patients has not yet been determined. The aim of this study was to understand the role of free flap reconstruction after skin cancer treatment, taking into account margin status assessment, recurrence and metastasis rates, and survival outcomes in patients with MSCs and NMSCs, and to evaluate the literature data in order to assess the advantages and disadvantages, indications, and contraindications of this reconstructive technique.

## 2. Materials and Methods

This systematic review adhered to the recommendations of the preferred reporting items of systematic reviews and meta-analysis (PRISMA) 2009 guidelines (Supplementary Materials) [16,17]. A computerized MEDLINE search was conducted utilizing the PubMed database of the U.S. National Library of Medicine (https://pubmed.ncbi.nlm.nih.gov/), encompassing studies published from January 2004 to May 2024. Three authors independently screened the articles and selected and extracted data pertaining to malignancy characteristics, reconstructive techniques, outcomes, and complications.

### 2.1. Search Strategy

Search strings were generated by combining tumour-related and treatment-related keywords by using the Boolean operators “AND”. Tissue or treatment-related keywords themselves were combined with the function “OR”. The keywords used were proven for MeSH terms.

**Figure 1:**
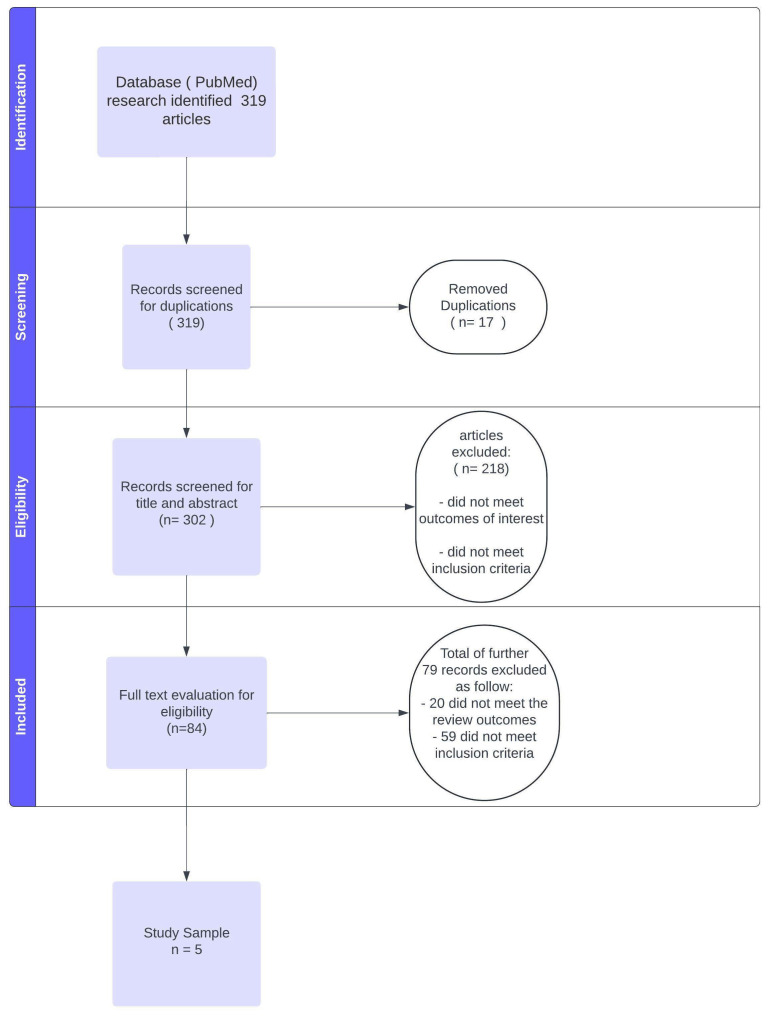
PRISMA flowchart showing the selection process.

The a priori set of inclusion criteria comprised randomized controlled trials, controlled clinical trials, controlled trials, and systematic reviews, with English full-text availability, specific histotypes of primary or recurrent skin cancer (squamous cell carcinoma, basal cell carcinoma, melanoma), and studies including a minimum of 15 cases. All articles encompassing cases pertaining to other skin or soft tissue neoplasms were excluded. Publications that incorporated post-traumatic cases in their reports were admitted when oncological findings and outcomes were independently evaluated. Exclusion criteria encompassed the pediatric population, benign skin lesions, mesenchymal origin of the malignancy, mucosal or uveal MSCs, NMCSs, and studies in which the nature of the excised tumor could not be clearly determined. Studies that incorporated nonhomogeneous etiologies of defects were included only if each outcome could be extrapolated from individual patients and neoplasms. Subsequently, oncological and clinical data, including previous interventions, adjuvant and neoadjuvant therapies, nodal status, distant metastasis, and follow-up time, were recorded when available. Surgical outcome parameters such as healing time, flap survival, revision rate success, and minor and major complications were also documented. Titles and abstracts of the articles were independently screened by three researchers (F.M., R.C., S.L.), and the articles were selected for full-text revision by two researchers (F.M., T.B.) independently. The PRISMA flowchart representing the collection and selection of articles is shown in Figure 1.

### 2.2. Data Extraction and Quality Assessment

Data were manually extracted from the included studies by two researchers (F.M. and R.C.) independently of each other. In case of disagreement, a third researcher (S.L.) checked the variables, and consensus was reached through discussion. Two researchers independently evaluated 79 full-text studies (F.M. and T.B.). A third researcher (S.L.) rated it in case of disagreement. Owing to the nature of the studies and the heterogeneity of the reported data selected in the review, a robust statistical analysis was not feasible. Therefore, quality assessment of the evidence from the individual studies included in the selected publications was conducted. The GRADE^®^ rating [18] was applied to the results to present summaries of the evidence and provide a systematic approach and quality assessment.

## 3. Results

A total of 319 studies were screened to eliminate duplicate and out-of-topic publications. Ultimately, 84 articles underwent comprehensive evaluation, with only 5 meeting the inclusion criteria. Notably, the cut-off of a minimum of 15 cases per study had the most significant impact on reducing the review sample. This threshold was deemed necessary to mitigate the potential influence of anecdotal reports.

Table 1 presents a comprehensive overview of the oncological findings derived from the studies included in the review such as tumor histology, location, margin status of tumor excision, and presurgical therapies administered. However, it is important to note that some studies had incomplete data, particularly regarding margin status after excision and details of adjuvant therapy [19].

The reporting of survival outcomes varied across the studies, reflecting the heterogeneity in follow-up periods and outcome measures. While some studies reported overall survival without specifying the time frame [20], others provided more detailed information on two-year and five-year overall survival rates. The mean follow-up times ranged from 19.9 to 31.5 months, indicating a relatively short-term assessment of outcomes [21,22]. Notably, one study [23] only reported median survival, further emphasizing the inconsistency in outcome reporting.

The findings pertaining to the flap utilized for reconstruction and the duration of hospital stay are presented in Table 2.

It was not feasible to extract data on wound healing time; however, it appeared to be roughly consistent with the hospital stay duration. Minor and major complications have been documented, along with the success rates of salvage procedures. Regarding this final aspect, it is noteworthy that in only two studies [20,21], a major complication, defined as a potential cause of flap failure, was addressed through a salvage procedure. In two additional studies [19,23], despite the occurrence of severe complications, no indication for surgical reintervention was provided.

**Figure 2:**
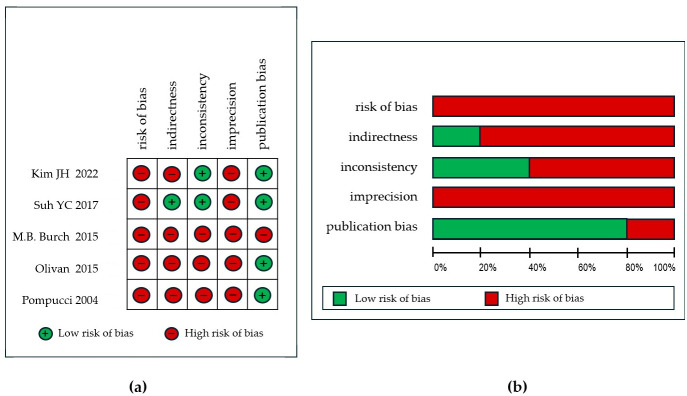
(**a**) Illustrative representation of the risk of bias among the studies included in the review; (**b**) the overall incidence of each type of bias [19,20,21,22,23].

Finally, the selected studies’ quality assessment with GRADE rating is summarized in Table 3 and in Figure 2.

## 4. Discussion

The cancer-related features of the studies included in the analysis exhibited significant diversity in the parameters examined and documented, making it difficult to draw comparisons and comprehensive conclusions. Kim et al. [20] report on melanoma patients, providing detailed data on histology, lymph node involvement, and recurrence. Sentinel lymph node biopsy (BLS) was performed in all patients, detecting regional metastasis in 10 out of 21 cases. Distant metastases were excluded via PET-CT, ensuring that the cohort included only patients without systemic spread. With a mean follow-up of 18 months, regional recurrences were reported in 2 cases and distant metastases in 3 cases, despite a 100% overall survival rate. The study population comprised patients with clinical stage 0-II, with more than 70% exhibiting no nodal or distant metastasis. All metastases occurred in SLNB-positive patients, consistent with the initial clinical stage. From this perspective, microsurgery does not appear to have influenced prognosis, given that the R0 margin obtained in all procedures is positively correlated with no recurrence of disease at the primary cancer site; however, the considerably limited follow-up precludes definitive conclusions. Suh et al. [21] also examined melanoma patients, reporting positive lymph node involvement in four cases. The study provided detailed follow-up data, with a median duration of 31.5 months, and presented comprehensive survival outcomes. Five-year overall survival was 73.2%, while progression-free survival was 44%, with local recurrence plateauing at 22.2% after 34.5 months. Distant metastases occurred in 36% of cases. Most patients (n = 43, 72.9%) had a histopathological diagnosis of the acral lentigenous type. The remaining patients were diagnosed with the lentigo maligna type (n = 4, 6.8%), and only 12 patients were diagnosed with the nodular type (30.3%). With respect to melanoma thinness (60.9% with thickness < 3 mm), the margins were widely clear (65.2% longer than 2.5 cm), which partially mitigated the high risk of local recurrence in the acral lentigenous subgroup of patients; however, compared with the literature [24,25], this study showed a decreased 5-year free survival of 54.3–64.5%. The authors explained this adverse survival, arguing that only large and advanced melanomas were included because they required a reconstruction option such as free flap, but the majority of melanomas were classified as stage 0-II (94%); in the absence of data about the size of the primary tumors, this statement seems not to be solid. Burch et al. [22] focused on basal cell carcinoma (BCC), reporting negative lymph node involvement and no distant metastases in all patients. The study provided survival data with overall disease-specific survival at 69% and a two-year disease-free survival rate of 72%. Recurrence occurred in six patients (five local, one distant), with a mean time to recurrence of 14 months. The study population comprised high-risk BCCs (15.7% of cases had one high-risk feature documented; 84.2% exhibited > 1 high-risk factor), with perineural invasion present in 13.2% and bone involvement in 44.7%; consequently, prior or adjuvant radiotherapy was administered to 55.3% of patients. However, no statistically significant advantage was demonstrated in disease-free and overall survival. This outcome may be attributed to the limited cohort size, which potentially does not represent a statistically robust sample, and the relatively short mean follow-up period. For the same reasons, the potential impact of microsurgical reconstruction cannot be ascertained. Pompucci et al. [23] included recurrent head and neck SCCs and BCCs, all cases of locally advanced tumor (T4) which invaded either the anterolateral or posterolateral skull base, with lymph node involvement documented in 4 of 18 patients (22.2% staged as N1). The mean follow-up period was 25 months and the median survival time was 32 months. In this study, the administration of adjuvant radiotherapy was found to be statistically significant (*p* = 0.016) in improving the median survival time (32 months for the 10 patients who received radiotherapy and 12 months for the eight patients who did not receive radiotherapy). Globally, the survival data demonstrated a significantly (*p* = 0.0007) longer survival time for patients with no residual disease; on the other hand, surgical treatment did not seem advisable for patients affected by T4N1 SCC with extensive invasion of the petrous bone, orbital apex, infratemporal fossa, and deeper structures of the neck. In similar cases, consistent residual disease is invariably correlated with poor prognosis.

Due to the heterogeneity of the collected data, encompassing various histotypes, stages, therapies and the overall limited sample size, true stratification of the findings is unattainable. Consequently, most researchers have confined their studies to a single type of skin cancer. However, numerous other factors, such as population selection, impede the comparison of survival and flap success rates.

Regrettably, all studies were characterized by a notably limited follow-up period, which introduces a potential bias concerning the reported survival rates. Kim et al. [20] documented a 100% survival rate in a cohort affected by acral melanoma, with nodal involvement present in 47.6% of cases; this outcome appears unduly optimistic. However, the median follow-up duration was 18 months. Such a brief timeframe precludes definitive conclusions regarding tumor-related and disease-free survival. Conversely, Burch et al. [22] reported a stratification of survival rates in the BCC population, decreasing from 80% to 66% at 2-year and 5-year follow-ups, respectively. This finding is unexpected given the limited metastatic propensity of basal cell carcinoma, yet the specific tumor location (head and neck) may be associated with aggressive histotypes, which can rapidly invade deeper layers, bone, and ultimately the brain, leading to patient mortality. The aforementioned confounding factors may have resulted in erroneous conclusions when compared without a detailed clinical condition description.

Another challenge is the forthcoming application of promising therapies, including immunotherapy, targeted therapy, and Hedgehog pathway inhibitors, in novel strategic combined settings. Immunotherapy is likely the most effective treatment for advanced squamous cell carcinoma (SCC), both locally advanced and metastatic, with ongoing trials assessing its impact in a neoadjuvant setting prior to surgical intervention. In melanoma, immunotherapy and BRAF-targeted therapy have demonstrated reliable positive outcomes; however, their current use is limited to an adjuvant setting or as an alternative for unresectable cancers. Finally, the role of Hedgehog pathway inhibitors in locally advanced and metastatic basal cell carcinoma (BCC) has been investigated, with some authors reporting positive and concrete results. It is important to note that this therapy requires continuous administration, as it involves the suppression of oncogene promoters; once discontinued, the affected cells are prone to degeneration. Therefore, its application in a conventional neoadjuvant setting to reduce cancer extension and transition the stage from unresectable to resectable is presently unfeasible.

Regarding the quantity of oncologic recordings, fewer reconstruction parameters were examined, which appears inconsistent with the nature of studies focused on microsurgery. While the efficacy of microsurgical tissue transfer in skin cancer patients has been established, data on healing duration and patient quality of life remain insufficient. Functional outcomes are particularly relevant in free flap reconstruction, as they directly influence the patient’s ability to resume daily activities and overall life satisfaction. Although some studies have reported minor parameters such as wound healing duration or complication rates, comprehensive assessments of functional recovery, psychological adaptation, and patient-reported outcomes are infrequently prioritized. Kim et al. [20] reported outcomes for 21 patients, primarily elderly males (average age 67.7 years), who underwent anterolateral thigh (ALT) flap reconstruction. The average hospital stay was 15.3 ± 6.4 days. Minor complications, including one case each of lymphedema and marginal wound disruption, were rare. Major complications requiring revision surgery occurred in three patients, all successfully salvaged. Suh et al. [21] evaluated 59 patients undergoing reconstructions with ALT, superficial circumflex iliac artery perforator (SCIP), posterior interosseous artery perforator (PIAP), upper medial thigh (UMT), and superior gluteal artery perforator (SGAP) flaps. Minor complications, such as hematomas and wound issues, were limited to six cases and resolved conservatively. Nine patients required revision surgery for vascular compromise or hematomas, with a high salvage rate. Although there were two cases of complete flap loss, the overall success rate was 77.7%, which underlines the effectiveness of the reconstructive approach. An analysis of the average time from surgery to weight-bearing ambulation was conducted based on the specific location of the foot melanoma. In the non-weight-bearing surface group, the mean ambulation period was 9.3 days, while in the weight-bearing surface melanoma group, it was 13.74 days. The difference between these groups was found to be statistically significant (*p* < 0.0001). Long-term functional assessment was performed via telephone survey for patients at least one year post-operation using the Visual Analog-Scale Foot and Ankle (VAS FA) [26], yielding an average total score of 94.1. All patients reported minimal complications in their functional status. However, the categories of foot pain during activity and sensation reduction were ranked as the two worst, with average scores of 83.9 and 74.8, respectively. These outcomes suggest that a free flap is not only a reliable option but may also lead to quicker recovery, contrary to previous beliefs that complex procedures require longer healing times. These findings could be particularly beneficial for elderly patients, as delays in ambulation and rehabilitation can result in extended recovery periods. Early and continued ambulation plays a crucial role in enhancing life expectancy and quality of life [27]. Burch et al. [22] reported on 38 cases involving radial forearm free flap (RFFF), transverse rectus abdominis myocutaneous (TRAM), latissimus dorsi, and ALT flaps. Hospitalization was notably shorter, averaging 5.24 days. While 37% experienced minor complications, including hematomas, infections, and osteoradionecrosis, these did not significantly impact the final reconstructive outcomes. In a smaller cohort of 22 patients, Olivan et al. [19] documented outcomes using ALT chimeric flaps. Complications were minimal, with only two cases of partial flap necrosis successfully treated with negative pressure wound therapy, and no major complications. In the postoperative follow-up period, the shape and stability of the flap were examined clinically, along with an evaluation of the patient’s walking progress and any postoperative complications. Sensory thresholds of the skin were measured in grams per square millimeter using an instrument with a pressure transducer capable of converting pressure into an electrical signal. Flap viability was complete in 23 patients, and 2 patients experienced complications with partial flap loss of the cutaneous component, necessitating surgical debridement 14 days post-operatively. These wounds subsequently healed by secondary intention, facilitated by negative pressure therapy. At 12 months post-operatively, following reoperation, flap contour was assessed as satisfactory in all patients, and flap stability was determined to be restored. Instrumental examination and static one-point and moving one-point tests were conducted 12 months post-operatively in seven patients who underwent neurorrhaphy, on the skin of the ALT flap, and on the contralateral thigh skin (corresponding to the donor area). The authors concluded that the differences were statistically significant. Finally, Pompucci et al. [23] analyzed 18 cases primarily utilizing the latissimus dorsi and rectus abdominis muscles. Although complications included transient cerebrospinal fluid leaks and facial nerve palsy, these were associated with tumor eradication rather than flap selection; consequently, the microsurgical approach could not have influenced them. Furthermore, late complications such as radionecrosis and tumor recurrence do not appear to diminish the efficacy of these techniques; the free flaps survived and demonstrated efficacy in all patients, although in one case, a re-exploration for vascular failure was necessary. Notwithstanding the utilization of diverse flap types tailored to specific clinical scenarios, the complication rate remains low, and the selection of flap does not appear to influence the overall efficacy of reconstruction. This consistency across studies underscores the adaptability of contemporary reconstructive methodologies and their capacity to achieve reliable outcomes, irrespective of the primary tumor, stage, and anatomical location. In this review, the GRADE rating was applied due to the heterogeneity of the included records. By systematically evaluating the evidence quality for each outcome and summarizing the findings, this approach allowed for a systematic evaluation of these limitations and strengths. Suh et al. [21] conducted a nonrandomized study with follow-up data averaging 31.5 months. Although adherence to follow-up was implied to be complete, no explicit description of withdrawals or exits was provided. The data collection process was rigorous; however, the study lacked stratification for survival outcomes or histological subtypes, limiting the applicability of the findings. Moreover, the rationale for flap selection was not elucidated or correlated with specific outcomes. The moderate grade of evidence certainty has been established due to constraints in addressing population diversity and flap choice. Kim et al. [20] encountered comparable methodological limitations, including a nonrandomized and nonblinded study design that introduced inherent bias. The investigation uniformly applied ALT flaps, irrespective of variations in plantar defect size and depth, thereby introducing indirectness into the evidence. While inconsistency was not a significant concern, the variability in follow-up durations diminished the precision of the results. The absence of data correlating survival and progression-free survival restricted the broader applicability of the findings; thus, a moderate quality of evidence was attributed. Burch et al. [22] presented findings with substantial limitations, including a lack of outcome stratification by flap type and insufficient explanation for the variability in follow-up. Although recurrence was associated with histological subtypes, the study failed to stratify outcomes accordingly, introducing both bias and indirectness. The absence of a clear rationale for flap selection and the wide range of follow-up periods further contributed to the imprecision. These factors resulted in a very low GRADE score. The investigation conducted by Olivan et al. [19] exhibited several methodological limitations. Primarily, the study lacked randomization and stratification of outcomes and failed to categorize complications comprehensively. Furthermore, only seven patients who underwent neurorrhaphy were assessed for sensitivity recovery, introducing substantial inconsistency and indirectness biases. These significant methodological issues resulted in a very low evidence rating for this study. Pompucci et al. [23] discussed flap selection and survival outcomes, but did not categorize complications based on flap type. Bias was introduced by the nonrandomized design and the loss of follow-up, and the variability in follow-up times reduced precision. Despite these limitations, the study’s emphasis on survival outcomes provided valuable insights, which partially mitigated the low GRADE for the overall evidence. In summary, while the studies collectively contribute to understanding reconstructive outcomes, their methodological weaknesses, including biases, lack of stratification, and imprecision, significantly limit the quality of evidence. The absence of consistent reporting of primary clinical endpoints, including disease-free survival (DFS), overall survival (OS), and progression-free survival (PFS), impedes the ability to ascertain whether reconstruction influences survival outcomes. Moreover, the treatments administered before surgery are frequently inconsistently documented. This information is essential to evaluate the reconstructive outcomes, as prior interventions can significantly affect tissue quality, complicate flap survival, and alter post-op recovery. For instance, patients who have undergone radiotherapy may exhibit poorer wound healing or require specific flap choices, impacting on complication rate and quality of life. The inclusion criteria established in this review resulted in the exclusion of numerous articles due to several key factors related to the heterogeneity in cancer histotypes, etiologies, and focus areas of the existing studies. Multiple investigations have examined other cancers with distinct biological behaviors and prognostic implications, such as sarcomas, mucosal cancers, and dermatofibrosarcomas. These malignancies exhibit variations in aggressiveness, recurrence risk, treatment response, and planning, which could introduce substantial variability in the analysis. By restricting inclusion to the three selected skin cancer types, this review aimed to create a more homogeneous study population, thereby facilitating clearer comparisons and more precise conclusions regarding microsurgical reconstruction in the context of skin cancer. However, this methodological approach broadly limits the range of studies suitable for inclusion, thus affecting the sample size and the feasibility of solid statistics. The low-rating GRADE scores reflect substantial limitations in study design, including insufficient sample sizes, retrospective methodologies, and heterogeneity in data collection approaches. These factors contribute to the diminished reliability and generalizability of the findings. A significant proportion of the reviewed articles failed to implement a standardized protocol for data collection and reporting, thereby compromising the quality and coherence of the collective body of evidence. Consequently, the synthesis of findings into meaningful recommendations for clinical practice remains problematic.

## 5. Conclusions

This systematic review highlights the extensive use of microsurgery for reconstruction after skin cancer excision; however, despite a growing body of work on microsurgical reconstruction, the literature remains limited by inconsistent reporting of oncological outcomes and the lack of a standardized approach to evaluate the impact of free flap reconstructions on both immediate and long-term cancer-specific results. Future studies should systematically report oncological variables and apply consistent outcome measures, such as disease-free survival, overall survival, and quality-of-life indicators, to allow for a clearer assessment of the benefits and drawbacks of microsurgery in an oncological context.

## Figures and Tables

**Table 1:** Tumor-related findings.

First Author, Year	Population	Histology	Location	N+	M+	Margins Status	Adjuvant Radio-Therapy	Adj. Chemo or Immuno Therapy	Neo-Adjuvant Therapy	Mean Follow Up (mo.)	Survival	Local Recurrence	Metastasis ^c^
Kim JH [20], 2022	21	Melanoma	heel	10	0	R0	0	5	0	18	100% (overall surv.)	/	23.8%
Suh YC [21], 2017	59	Melanoma	foot and ankle	4	0	R0	8	7	0	31.5	73.2% (5-year overall surv.) 44% (progression-free surv.) 51.4 months (median progression-free surv. with 95% CI: 17.2–85.6 months)	22.2%	36%
M.B. Burch [22], 2015	38	BCC	head and neck	0	0	/	17	0	4 (10.5%) ^b^	19.9	80% (2-year overall surv.) 66% (5-year overall surv.) 69% (overall disease-specific surv.) 72% (2-year disease-free surv.)	13%	2.6%
Olivan [19], 2015	22	Melanoma	plantar region	/	/	/	/	0	/	24	/	/	/
Pompucci [23], 2004	18	SCC (8) BCC (10)	head	4	/	R0 (44.4%) R1 (33.3%) R2 (22.2%)	10 ^a^	/	1 (5.5%) ^b^	25	median survival time 32 months (the lower 95% confidence limit 23 months)	11%	/

^a^ Adjuvant radiotherapy was recommended in 14 cases but was fully administered only in 10 (6 BCCs and 4 SCCs) because of two refusals and two deaths; ^b^ Neoadjuvant radiotherapy; ^c^ Nodal involvement or/and distant metastasis occurring after surgery.

**Table 2:** Reconstruction-related findings.

First Author, Year	Population	Type of Flap (no.) (%)	Mean Hospital Stay (Days)	Minor Complications ^a^	Major Complications ^a^	Flap Loss	Salvage Procedure (Success %)
Kim JH [20], 2022	21	ALT (21) (100%)	15.3 (±6.4)	lymphedema (1) marginal disruption (1)	venous congestion (3) hematoma (1)	0	4 (100%)
Suh YC [21], 2017	59	ALT (22) (36.6%) SCIP (30) (50%) PIAP (1) (1.6%) UMT (4) (6.6%) SGAP (3) (5%)	17.1	hematoma (3) seroma (1) wound infection (1) wound dehiscence (1)	arterial insufficiency (4) venous congestion (2) hematoma (3)	2 (complete) 2 (partial)	9 (77.7%) ^b^
M.B. Burch [22], 2015	38	RFFF (25) (65.7%) TRAM (6) (15.7%) Latissimus dorsi (4) (10.5%) ALT (3) (7.8%)	5.2 (range 3–11)	exposed bone (4) hematoma/seroma (4) surgical site infection (3) osteoradionecrosis (2) donor site evisceration (2)	0	0	0
Olivan [19], 2015	22	ALT (22) (100%)	/	0	flap necrosis (2)	2 (partial)	0 ^c^
Pompucci [23], 2004	18	Latissimus dorsi (15) (83.3%) TRAM (3) (16.7%)	/	/	radionecrosis (1)	1 (partial)	1 (100%)

^a^ Complications related to flap reconstruction and/or wound healing; ^b^ Two procedures were considered successful despite partial loss of the flaps; ^c^ There has been no mention of a salvage procedure, only late reinterventions for flap contour improvement.

**Table 3:** GRADE of evidence rating.

First Author, Year	Population	Study Design	Risk of Bias	Indirectness	Inconsistency	Imprecision	Publication Bias	Quality of Evidence
Kim JH [20], 2022	21	retrospective	very serious ^a,b^	serious ^c,d^	not serious	very serious ^e^	undetected	moderate
Suh YC [21], 2017	59	retrospective	serious ^a^	not serious	not serious	serious ^f^	not serious	moderate
M.B. Burch [22], 2015	38	retrospective	very serious ^a,b^	serious ^g^	serious ^h^	serious ^f^	Serious ^k^	very low
Olivan [19], 2015	22	retrospective	very serious ^a,b^	very serious ^i^	very serious ^j^	serious ^f^	not serious	very low
Pompucci [23], 2004	18	retrospective	serious ^a^	serious	very serious ^j^	serious ^f^	not serious	low

^a^ Performance and detection bias was not assessed blindly; ^b^ No information about the reason for follow-up loss/variability; ^c^ No functional outcomes stratification is available for each flap efficacy for weight-bearing and non-weight-bearing areas; ^d^ Survival or progression-free survival is not stratified for any flap or T stage; ^e^ The median progression-free survival had a 95% confidence interval of 17.2–85.6 months; ^f^ N Wide follow-up range; ^g^ P No recurrence stratification based on histology; ^h^ W Lack of stratification of outcomes per single flap; ^i^ N Outcomes evaluated for different patients differ; ^j^ L Significant between-study heterogeneity was detected in the overall analysis; ^k^ O The article demonstrates significant publication bias as it is funded by the National Institutes of Health and the National Cancer Institute, raising concerns about potential conflicts of interest or selective reporting.

## Data Availability

Data set was not created.

## Supplementary Materials

The following supporting information can be downloaded at: https://www.mdpi.com/article/10.3390/cancers17142371/s1.

## Abbreviations

The following abbreviations are used in this manuscript

MSCs	Melanoma skin cancers
NMSCs	Non-melanoma skin cancers
BCC	Basocellular carcinoma
SCC	Squamocellular carcinoma
